# Influence of Genetically Modified Human Umbilical Cord Blood Mononuclear Cells on the Expression of Schwann Cell Molecular Determinants in Spinal Cord Injury

**DOI:** 10.1155/2018/4695275

**Published:** 2018-02-18

**Authors:** L. R. Galieva, Y. O. Mukhamedshina, E. R. Akhmetzyanova, Z. E. Gilazieva, S. S. Arkhipova, E. E. Garanina, A. A. Rizvanov

**Affiliations:** ^1^Kazan Federal University, Kazan, Russia; ^2^Kazan State Medical University, Kazan, Russia

## Abstract

Spinal cord injury (SCI) unavoidably results in death of not only neurons but also glial cells. In particular, the death of oligodendrocytes leads to impaired nerve impulse conduction in intact axons. However, after SCI, the Schwann cells (SCs) are capable of migrating towards an area of injury and participating in the formation of functional myelin. In addition to SCI, cell-based therapy can influence the migration of SCs and the expression of their molecular determinants. In a number of cases, it can be explained by the ability of implanted cells to secrete neurotrophic factors (NTFs). Genetically modified stem and progenitor cells overexpressing NTFs have recently attracted special attention of researchers and are most promising for the purposes of regenerative medicine. Therefore, we have studied the effect of genetically modified human umbilical cord blood mononuclear cells on the expression of SC molecular determinants in SCI.

## 1. Introduction

The compensation of demyelination is one of promising approaches to overcome consequences of spinal cord injury (SCI). It is experimentally confirmed that after injury, the Schwann cells (SCs) migrate from the peripheral nervous system towards the site of injury and participate in axon remyelination [1]. SC transplantation demonstrates high efficacy in restoring the conduction; however, in this case, neuroregeneration is limited due to their short-term viability [2]. A lot of studies have been devoted to this topic; at the same time, factors affecting the migration and maintenance of SC-migrant survival are understudied.

Besides, there are other assumptions which can explain the phenomenon of SC appearing in the area of SCI such as (1) oligodendrocytes start expressing SC markers, (2) possible SC differentiation from neural progenitors in situ cannot be ruled out [3–5], or (3) remyelinating SCs of the central nervous system originate from precursors of oligodendrocytes [6]. Moreover, at present, there is an urgent need to reconsider possible expression of molecular determinants, identified with the SCs in the intact spinal cord.

In addition to an injury of the CNS, the SC migration and the expression of their molecular determinants can be influenced by cell-based therapy. In a number of cases, it can be explained by the ability of implanted cells to secrete neurotrophic factors (NTFs) [6]. For example, the SCs are a target of a glial cell line-derived neurotrophic factor (GDNF), and this NTF maintains their migration and stimulates their myelination [7, 8], while а vascular endothelial growth factor (VEGF) enhances their proliferation [9].

Human umbilical cord blood mononuclear cells (hUCB-MCs), as an option of cell-based therapy, are quite promising due to their low immunogenicity, availability, and easy and safe isolation as well as the ability to sustain a long-term storage. In SCI, the introduction of hUCB cells into bloodstream reduces an inflammation, exerts a neurotrophic effect [10], reduces the expression of proapoptotic genes, and, as a result, maintains neuron survival [11]. Also, transplantation of genetically modified hUCB-MCs into the area of SCI was shown to promote tissue sparing, behavioral recovery, and axonal regeneration as compared to controls without therapy [12]. It has been established in a number of experimental models that hUCB cells produce some NTFs including a nerve growth factor and VEGF [13, 14], which can enhance the SC migration. Taking into consideration the objectives of gene and cell-based therapy, it seems relevant to evaluate the expression of SC molecular determinants within the area of SCI and possible effects of genetically modified hUCB-MCs on them.

## 2. Materials and Methods

### 2.1. Isolation and Adenoviral Transduction of hUCB-MCs

Umbilical cord blood was obtained from healthy full-term pregnant women with the gestational age 39-40 weeks in accordance with the protocol and standards of the Stem Cell Bank of the Kazan State Medical University. The study was approved by the Institutional Review Board of the Kazan State Medical University. Mononuclear blood cells were isolated, and adenoviruses generated as previously described [12, 15]. After purification, hUCB-MCs were maintained in a RPMI-1640 medium (Sigma, USA) supplemented with 10% FBS (Sigma), penicillin, and streptomycin (100 U/ml and 100 *μ*g/ml, respectively, Sigma). Immediately after isolation, hUCB-MCs were seeded in 10 cm culture dishes and transduced with recombinant adenoviruses Ad5-VEGF and Ad5-GDNF, or Ad5-EGFP (enhanced green-fluorescent protein) at an MOI of 10. The cells were incubated in a humidified atmosphere of 5% CO_2_ at 37°C for 12–16 h. Immediately, before the introduction, the cells were washed with a Dulbecco's phosphate-buffered saline (DPBS, Paneco, Russia).

### 2.2. Spinal Cord Injury and Experimental Groups

Thirty-three Wistar rats (weighing 250–300 g each; Pushchino Laboratory, Russia) were used for SCI in the present study. Rats were randomly assigned to four groups. hUCB-MCs were injected after SCI as previously described [12]. The groups were (1) hUCB-MCs transduced with recombinant adenoviruses encoding VEGF and GDNF having therapeutic effect (SCI hUCB-MCs + Ad5-VEGF + Ad5-GDNF, *n* = 12), (2) hUCB-MCs transduced with a recombinant adenoviral vector encoding EGFP (SCI hUCB-MCs + Ad5-EGFP, *n* = 10), and (3) without injection (SCI only, *n* = 11). This study was carried out with the approval of the Kazan State Medical University Animal Care and Use Committee (permit number 5 dated on May 27, 2014), and experimental protocols were consistent with the recommendations of the physiological section of the Russian National Committee on Bioethics. Animals were housed in clear plastic cages with food and water.

The rats were deeply anesthetized with an intraperitoneal injection of chloral hydrate (80 mg/ml, 0.4 ml per 100 g, Sigma). After skin incision, laminectomy at the T8 vertebral level was performed. The impact rod (diameter 2 mm, weight 10 g) of an impactor was centered above T8 and dropped from a height of 25 mm to induce SCI. Immediately following SCI, UCB-MCs were injected intraspinally into two points (5 *μ*l/injection; 1 × 10^6^ cells/5 *μ*l DPBS, 2 × 10^6^ cells per rat). The distance from each injection point to the injury center was 1 mm. The rostral and caudal points of injection were 0.5 mm displaced to the left and the right from the midline. Then the wound was sutured. After surgery, rats were given gentamicin (25 mg/kg, Omela, Russia) intramuscularly for 7 consecutive days to protect against infection. Bladders of the injured rats were manually emptied twice daily until spontaneous voiding occurred.

### 2.3. Immunofluorescence Analysis

Transverse tissue sections (20 *μ*m) were incubated with primary and secondary antibodies shown in Table 1. For immunofluorescence labeling, the sections were blocked with a 5% normal goat serum at room temperature (RT) for 1 hour. Then incubated overnight at 4°C with a primary Ab raised in distinct species. Prior to visualization, the sections were incubated with fluorophore-conjugated secondary Abs for 2 h at RT. 4′,6-Diamidino-2-phenylindole (DAPI) (10 *μ*g/ml in PBS, Sigma) was used for the counterstaining of nuclei. Coverslips were mounted on the slides with a mounting medium (ImmunoHistoMount, Santa Cruz). The sections were investigated under a LSM 780 Confocal Microscope (Carl Zeiss, Germany). All sections were imaged in the *z*-plane using identical confocal settings (laser intensity, gain, and offset). Measurements were obtained from longitudinal histological sections collected at 50 *μ*m increments extending from the contusion center (observed area 2 mm^2^) of the SCI.

### 2.4. Western Blotting

Total proteins were extracted from spinal cords by homogenization of tissue samples in a RIPA buffer (Sigma) containing protease and phosphatase inhibitors as previously described [12]. Protein concentrations were measured by Protein Assay BCA Kit (Thermo Scientific) with bovine serum albumin (BSA) as standard. Protein extracts (40 *μ*g) were separated with 4%–13% gradient sodium dodecylsulfate polyacrylamide gel electrophoresis (SDS-PAGE) and blotted to polyvinylidene fluoride membranes. After incubation with Krox 20, peripherin, P0, p75, and HRP-conjugated anti-mouse/anti-goat IgG (Sigma), blots were visualized with a mixture of 1.25 mM luminol, 146 mM p-coumaric acid, and 34% H_2_O_2_. The detection and analysis of immune complexes were performed by the Gel Doc XRS+ System (Bio-Rad, Hercules, CA, USA). Bands were quantified with the use of ImageJ version 1.46, and densitometric levels of proteins were normalized to *β*-actin.

### 2.5. Electron and Immuno-Electron Microscopy

For electron microscopy, ultrathin sections (transverse and longitudinal) mounted on copper grids (Sigma-Aldrich, USA, 200 mesh) were incubated with uranyl acetate and lead citrate for double contrast. The sections were examined under a transmission electron microscope Jeol 1200 SX (Tokyo, Japan). For immunoelectron microscopy, after dehydration, pieces of the spinal cord were embedded in LR-white (Sigma, USA). Ultrathin sections were mounted on nickel grids with a formvar film (Sigma-Aldrich, USA, 200 mesh) and blocked with TBSNGS-BSA-Tx100 (Tris-buffered saline (Tris 0.01 M, NaCl 0.15 M pH = 8.2), normal goat serum 10%, bovine serum albumin 0.2%, and Triton X-100 0.1%) for 1 h. After rinsing in TBS, the sections were incubated overnight at 4°C with anti-P0 (P0, Santa Cruz, 1 : 150) antibodies and then 10 nm gold-conjugated secondary Ab (Sigma-Aldrich, USA) for 1 hour at room temperature. To enhance visualization, silver (Silver Enhancer Kit, Sigma-Aldrich, USA) was deposited on colloidal gold. Then, the sections were double-contrasted with uranyl acetate for 20 min at 60°C and lead citrate for 10 min at room temperature; they were examined with transmission electron microscopy.

### 2.6. Real-Time PCR of mpz, pmp2, and pmp22

Total RNA of hUCB-MCs and the freshly isolated spinal cord in all groups were extracted using Yellow Solve reagents (Silex, Russia) according to the manufacturer's instruction. cDNA were analyzed using a CFX 96 Real-Time PCR System (Bio-Rad, Hercules, CA, USA). Each PCR reaction (15 *μ*l) contained 0.5 *μ*l cDNA, 2.5x reaction mixture B (Syntol, Russia), 200 nM of each primer, and the probe (100 nM) (Table 2). The PCR protocol conditions were as follows: preheating at 95°C for 3 min, followed by 39 cycles of 95°C for 10 s, and 55°C for 15 s including the plate-read. The gene changes in the groups were calculated by normalizing to 18S ribosomal RNA. The expression levels after SCI were set at 100%. All RT-PCR reactions were performed in triplicate.

### 2.7. Statistical Analysis

Data are presented as means ± standard deviation (SD). A Student's *t*-test and a one-way analysis of variance (ANOVA) with a Tukey's test or two-way analysis of variance (ANOVA) were used for multiple comparisons between all experimental and control groups. A value of *p* < 0.05 was considered statistically significant. The data were analyzed using the Origin 7.0 SR0 software (OriginLab, Northampton, MA, USA).

## 3. Results

### 3.1. Expression of Proteins Krox 20, Peripherin, р75, and P0 by Cells of the Intact Spinal Cord and the Sciatic Nerve

**Figure 1:**
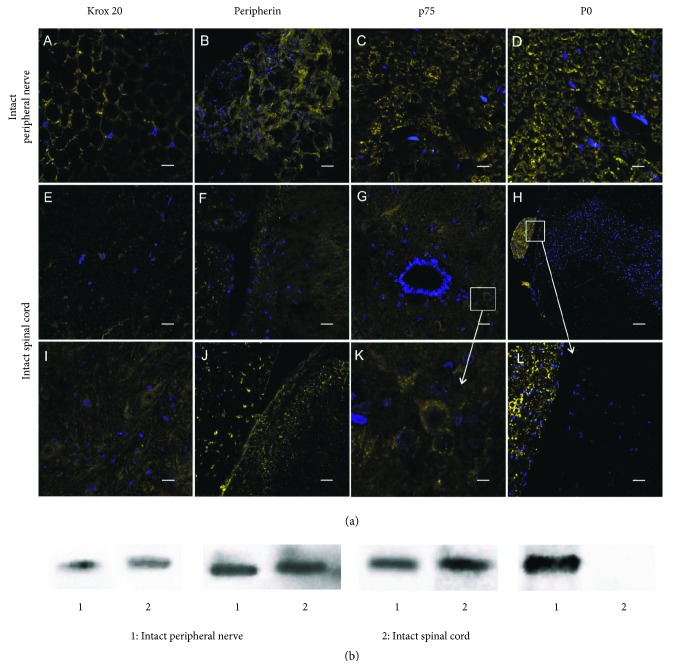
(a) Immunofluorescence analysis of SCs with antibody against Krox 20 (A, E, I), peripherin (B, F, J), P0 (C, G, K), and p75 (D, H, L) in an intact peripheral nerve and the intact spinal cord. P0^+^ cells were not detected in the intact spinal cord. Scale bar: 5 *μ*m (E, K), 10 *μ*m (A, C, D, F–J, L), 20 *μ*m (B). (b) Western blotting of Krox 20, peripherin, P0, and p75 proteins, respectively, in an intact peripheral nerve (1) and the intact spinal cord (2).

In order to determine specific SC markers, immunohistochemical staining for proteins Krox 20, Peripherin, р75, and P0 in an intact spinal cord and a peripheral nerve was performed. Based on the results obtained, there was an expression of all the proteins studied in the peripheral nerve. However, there were cells expressing Krox 20, Peripherin, and р75 only in the intact spinal cord (Figure 1(a), E–G and I–K). Krox 20^+^ cells were equally distributed in both the gray and the white matter. The expression of p75 was observed primary in the gray matter. Peripherin-expressing cells were detected within a dorsal root entry zone (DREZ).

We performed Western blotting to confirm the expression of proteins in the intact spinal cord and an intact peripheral nerve (Figure 1(b)). Our results showed that proteins Krox 20, Peripherin, and р75 were expressed in the intact spinal cord except for P0 protein. Nevertheless, all investigated proteins were detected in an intact peripheral nerve. Thus, we have demonstrated that P0 is the most specific marker for the SCs and can be used to visualize these cells after SCI.

### 3.2. Schwann Cell Distribution in the Area of Spinal Cord Injury in Experimental Groups

**Figure 2:**
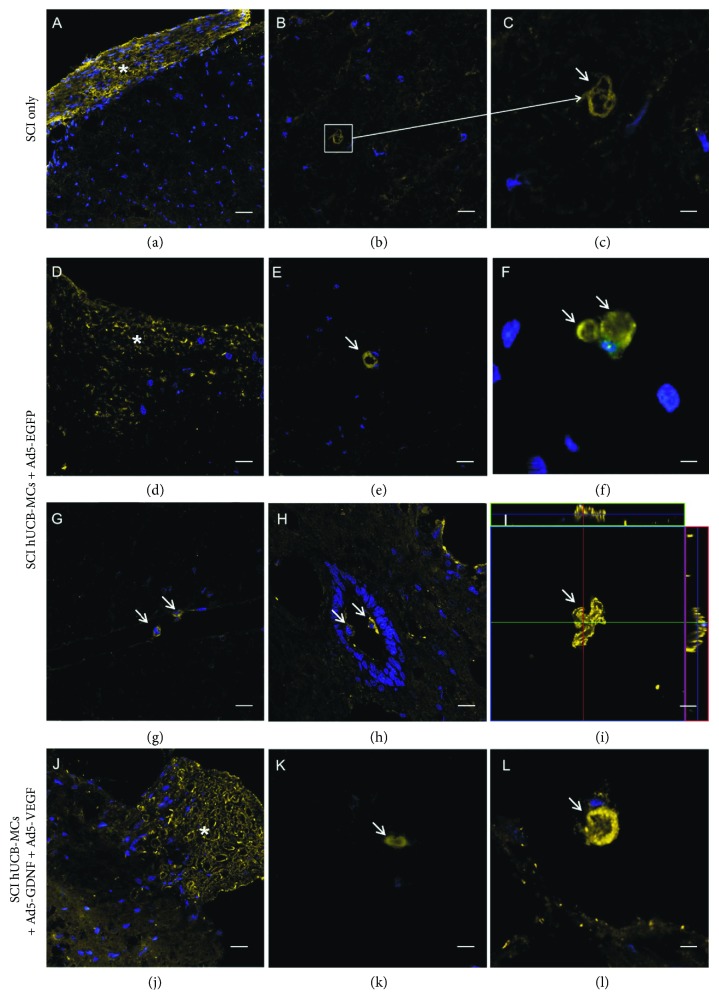
Immunofluorescence analysis of SCs with antibody against P0 protein in the area of SCI (a–c), SCI hUCB-MCs + Ad5-EGFP (d–i), and SCI hUCB-MCs + Ad5-GDNF + Ad5-VEGF (j–l) groups. P0^+^ cells were visualized in the DREZ (a, d, j), white matter (b–c, e, k), posttraumatic cavity (f, l), central canal (h), and fissura mediana anterior (g). (i) 3D-immunofluorescence visualization of the protein P0 in the white matter of the spinal cord in the hUCB-MCs + Ad5-EGFP groups on day 30 after SCI. SC are capable of myelinating several axons. Arrows and asterisks indicate P0^+^ myelin/cells (single cells) and P0^+^ area (clusters of cells), respectively. Scale bar: 20 *μ*m (a), 10 *μ*m (b, e, g–h, j–k), 5 (d, f, i, l), and 4 *μ*m (c).

Based on the results of immunohistochemistry on day 30 after SCI, there were axons with Р0^+^peripheral myelin in the white matter within the area of injury in all groups of animals (Figure 2). In the SCI, only group Р0^+^ myelin was detected in the gray matter primarily within the area of dorsal funiculi (DF) in the number of 2-3 axons with peripheral myelin over an area of 0.026 mm^2^, with its most part being disintegrated (Figures 2(b) and 2(c)).

In the groups implanted with hUCB-MCs, Р0^+^ myelin was also detected in the spinal gray matter both in intact tissue and in cavities of various diameter (Figures 2(d)–2(f), 2(k), and 2(l)). There were no statistically significant differences in the amount of Р0^+^ myelin in the white matter between experimental groups. However, there was much rarer myelin disintegration in the groups implanted with hUCB-MCs. In the experimental groups, Р0^+^ cells were also identified within the cavity of the central canal as well as in the anterior median fissure (Figures 2(g) and 2(h)).

We have found out that there was Р0^+^ myelin in the DREZ in the groups with transplantation of hUCB-MCs, while no Р0^+^ myelin detected in this area in the animals with SCI only (Figures 2(a), 2(d), and 2(j)). That the SCs myelinating several axons at the same time were detected within the area of injury in the animals of the hUCB-MC transplantation groups was an interesting finding (Figure 2(i)).

**Figure 3:**
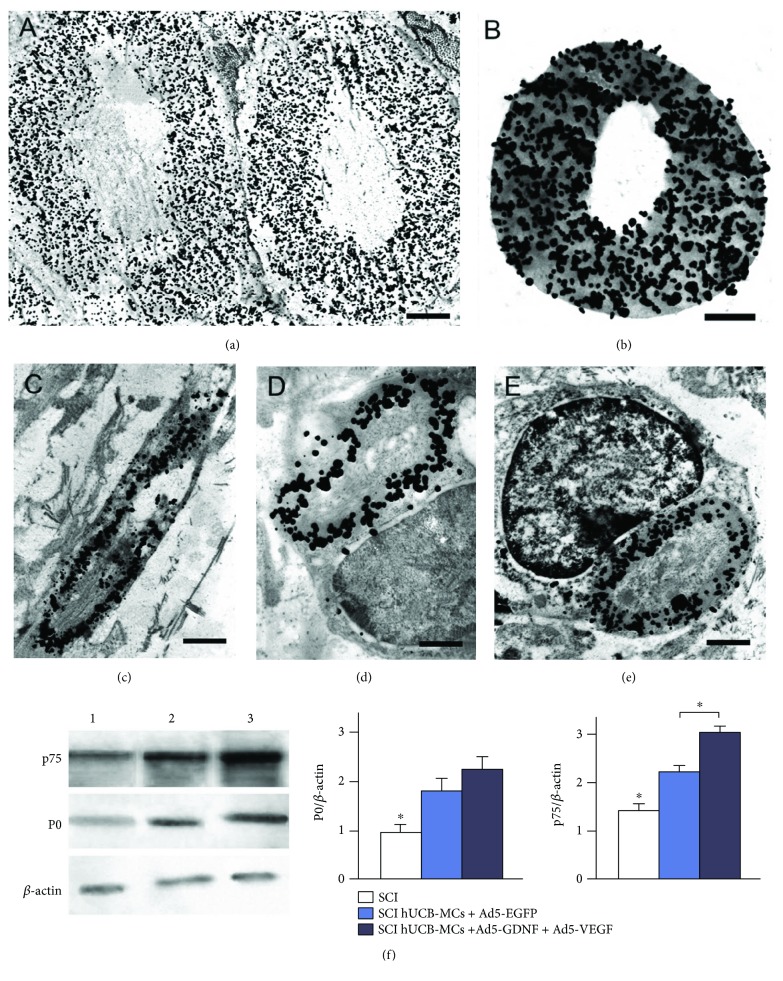
Immunoelectron microscopy results (a–e). P0^+^-positive reaction concentric myelin layers around the central projection in transverse (a-b) and longitudinal sections of myelin fibers (c). The SCs detected in the experimental groups contained a single myelin fiber which also had a specific precipitate (d-e). Scale bar: 1 *μ*m (a, c–e), 500 nm (b). (f) Western blotting of proteins p75 and P0 in the area of injury in the experimental groups (1: SCI only, 2: hUCB-MCs-Ad5-EGFP, 3: hUCB-MCs + Ad5-VEGF + Ad5-GDNF). Staining with Abs against p75 and P0 detected specific bands at 75 kDa and 28 kDa in tissue samples. *β*-Actin was used as a loading control. Densitometry analysis demonstrated a significant change in p75 and P0 levels relative to the *β*-actin expression after SCI. Differences were statistically significant between the SCI only and other experimental groups (^∗^*p* < 0.05). Differences in p75 level were also statistically significant between groups with injection of hUCB-MCs (^∗^*p* < 0.05). One-way ANOVA.

We have previously performed ultrastructural analysis of the injured spinal cord tissue after transplantation of native and genetically modified hUCB-MCs [16]. The results demonstrated that there are myelin-forming SCs within the area of injury in the experimental groups; they have an average electron density cytoplasm with a large irregular or bean-shaped nucleus containing heterochromatin and a basement membrane which covers the entire surface of a SC. These cells were identified in the SCI group at lower levels; most of them had features of apoptosis and myelin sheath disintegration by day 30 after injury as compared to the groups with hUCB-MC transplantation. The findings of immuno-electron microscopy confirm the presence of peripheral myelin in the experimental groups of animals (Figure 3). A positive response to the marker of peripheral myelin P0 can be inferred by the presence of a precipitate of colloid gold nanoparticles in concentric myelin layers around the central projection in transverse (Figures 3(a) and 3(b)) and longitudinal sections of myelin fibers (Figure 3(c)). The SCs detected in the experimental groups contained a single myelin fiber which also had a specific precipitate (Figures 3(d) and 3(e)); no response to P0 was observed in the cell cytoplasm and nucleus.

### 3.3. Analysis of P0 and p75 Expression

To evaluate relative expression of p75 and Р0, proteins were extracted from spinal cord tissue of the experimental group rats in the area of injury on postinjury day 30. Based on the results, there were proteins P0 and p75 in all experimental groups (Figure 3). It has been found out that the expression of both proteins after SCI and hUCB-MC transplantation significantly increases as compared to the SCI group without therapy (*p* < 0.05). However, there was a significant difference in the p75 expression between groups with hUCB-MC transplantation where the value was by 30.4% higher in the hUCB-MCs + Ad5-GDNF + Ad5-VEGF group than that in the hUCB-MCs + Ad5-EGFP group.

### 3.4. Analysis of *mpz*, *pmp2*, and *pmp22* mRNA Expression

To quantify the *mpz*, *pmp2*, and *pmp22* expression, mRNA isolated from intact spinal cord, an intact peripheral nerve, and the spinal cord tissue in the area of injury in the rats of experimental groups was used. Although protein products of these genes are highly specific for peripheral myelin, we have also detected low levels of the mpz, pmp2, and pmp22 mRNA expression in the CNS.

**Figure 4:**
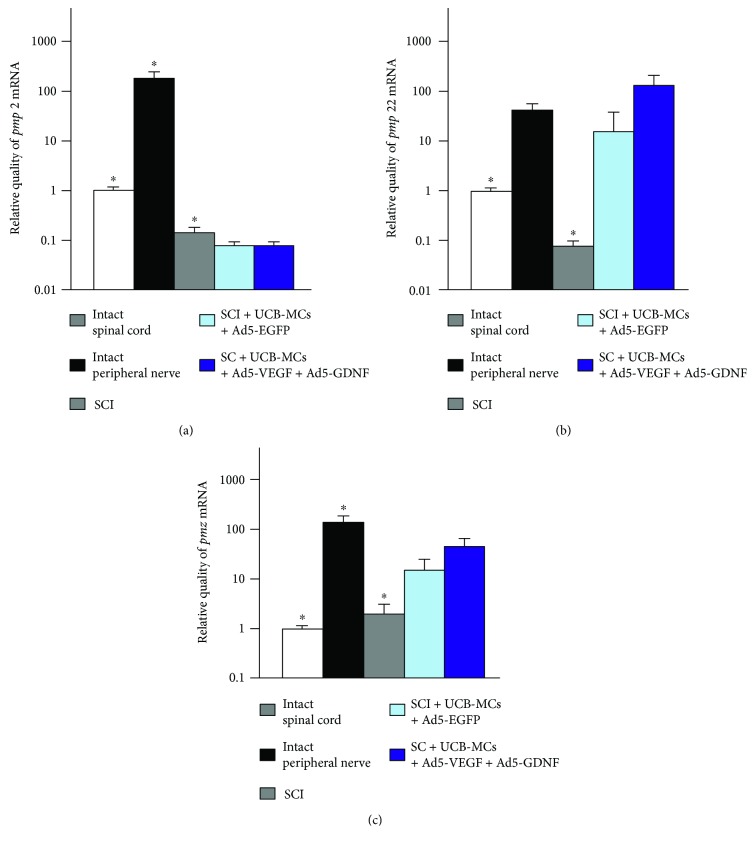
Relative expression of *pmp2*, *pmp22*, and *mpz* mRNA in the area of SCI in rats of the experimental groups. The levels of *pmp2*, *pmp22*, and *mpz* mRNA expression in the intact spinal cord were considered 100%. The RNA amount was normalized to 18 S. ^∗^*p* < 0.05 statistically significant difference between the group mentioned and other experimental groups.

According to the RT-PCR data, the *pmp2* mRNA expression decreases in all experimental groups as compared to the intact spinal cord (Figure 4(a)). There was a decreased expression of this gene mRNA in 5 and 12.5 times in the group of SCI without cell transplantation (*p* < 0.05) and in those with hUCB-MC transplantation (*p* < 0.05), respectively. At the same time, the expression level of *pmp2* mRNA was significantly higher in the SCI group when compared with the groups of hUCB-MC transplantation (*p* < 0.05).

With hUCB-MCs + Ad5-EGFP transplantation, the *pmp22* mRNA expression in the area of SCI increases in 31 and 128 times in the groups with hUCB-MCs + Ad5-EGFP (*p* < 0.05) and hUCB-MCs + Ad5-VEGF + Ad5-GDNF transplantation (*p* < 0.05), respectively, as compared to the same parameter in the intact controls. On the contrary, the expression of this gene decreased by more than 10 times in the group of SCI without cell transplantation as compared to the same parameter in the intact controls (*p* < 0.05).

RT-PCR has also shown that the *mpz* mRNA expression increases in all experimental groups as compared to the intact spinal cord (Figure 4(c)). The expression of this gene increases in 4 (*p* < 0.05), 29 (*p* < 0.05), and 59 (*p* < 0.01) times in the group of SCI without cell transplantation, the first control group (hUCB-MCs + Ad5-EGFP), and the experimental one (hUCB-MCs + Ad5-GDNF + Ad5-VEGF), respectively, as compared to the same parameter in the intact controls.

Thus, we have demonstrated that the genes which are highly specific for peripheral myelin differently respond to SCI and hUCB-MC transplantation that is confirmed by different levels of the mpz, pmp2, and pmp22 mRNA expression in the experimental groups.

## 4. Discussion

In this study, we have evaluated the SC population in the area of SCI when implanting genetically modified hUCB-MCs, having previously determined the most specific molecular determinants for the SCs and peripheral myelin in particular. The results of Western blotting and immunohistochemical staining of the intact spinal cord and a peripheral nerve confirmed the findings on high specificity of the protein Р0 for myelin-forming SCs, that is, peripheral myelin. However, there are studies which give evidence to the protein P0 presence in the intact spinal cord. Satto et al. (1999) explained it by the fact that in young rats (9-week old) P0 is nonglycosylated in the spinal cord, while it being glycosylated in adult rats (29-month old) [17]. These differences in various studies are therefore related to the specificity of an epitope in which the used antibodies bind that affects the detection of P0 in the spinal cord. Also, P0 was present in the dorsal columns 10 weeks after spinal cord injury [5].

Our study has demonstrated that the proteins Krox 20, р75, and peripherin can be expressed not only by the SCs in the peripheral nerve system (PNS) but also by cells of the gray and white matter of the intact spinal cord whose group specificity is to be determined. Although the abovementioned proteins are considered specific for the SCs, Krox 20 is a transcription factor promoting the transition of immature SCs to myelinating ones; the protein р75 is specific for SC precursors, while peripherin is widely expressed in the cell body and axons in the PNS [18, 19]. Nevertheless, some authors have the results which are in line with ours. In particular, an intermediate filament peripherin is expressed in neurons [20], p75 was detected in the dorsal horn of the intact spinal cord [21], and Krox 20 is localized in Me5 (mesencephalic trigeminal) neurons [22].

It has been experimentally established that SCs can be involved in remyelination of axons of the CNS and, therefore, promote regeneration [23–25]. Yet, myelin of the SCs is functional as it restores the conduction of axons. The SCs forming substitutive peripheral myelin are gradually replaced with oligodendrocytes [26]. In addition, dedifferentiated SCs actively produce neurotrophic factors, extracellular matrix, and adhesion molecules, and other molecules associated with axonal growth stimulate processes of neuroregeneration more aggressively [27–29]. Endogenous SCs migrate not only in an injury epicenter but also rostrally and caudally from the epicenter usually moving along a cavity in the site of injury [30] that is consistent with the results of our study.

Signals which trigger mechanisms of SC migration into the injured CNS are presently still unknown [31]. However, it is noted that SC migration is significantly influenced by NTFs. The SCs have been shown to be targets for GDNF, with this NTF maintaining their migration and stimulating myelination [7, 8, 32]. NGF, a ligand of the neurotrophic р75 receptor, promotes the migration of immature р75-expressing SCs [33]. NT-3 was demonstrated to stimulate SC migration through a Trk receptor. The interaction of BDNF and p75^NTR^ enhances the process of myelination; however, it has an inhibiting effect on the SC migration [34].

It has been established that SC migration can be influenced by different modes of direct and cell-mediated delivery of NTF genes. We have previously shown that transplantation of hUCB-MCs, transduced by an adenovirus, carrying the gene *gdnf* and a direct delivery of adenoviral vectors encoding a similar gene promotes SC migration to the spinal cord [15]. By the third week after SCI, the transplantation of genetically modified fibroblasts carrying the gene *bdnf* contributed to the significant increased SC number in the area of injury [35]. With a plasmid gene delivery, an increased VEGF level in the injured nerve maintains and enhances the growth of regenerating nerve fibers, by combined angiogenic, neurotrophic, and neuroprotective effects, and increases the SC proliferation [36]. We have previously shown that the plasmid delivery of the genes *vegf* and *fgf2* into an injured spinal cord leads to the increased SC number in the area of SCI [37].

Our results demonstrate a positive effect of hUCB-MC transplantation on the migration potential (detection of Р0^+^ myelin in the DREZ) of the SCs and the integrity of peripheral myelin. Based on the immunohistochemical and Western blotting findings, there were no significant difference in the protein Р0 expression between hUCB-MCs-Ad5-EGFP and hUCB-MCs + Ad5-VEGF + Ad5-GDNF groups. Thus, it can be concluded that the subpopulation of SCs with a P0^+^ phenotype in the area of SCI seems to be a target of the cell transplant factor supporting effect, rather than the delivery of therapeutic genes. A previously demonstrated early increase of the SC number in the area of SCI with a separate delivery of the genes *vegf* and *gdnf*, does not exert a similar effect in case of a combined delivery of these genes by means of hUCB-MCs. This in turn suggests not only the lack of a synergistic effect of *vegf* and *gdnf* on the SCs but also possible antagonism from these NTFs to the SCs.

That SCs simultaneously myelinating several axons within the area of injury in the groups with hUCB-MC transplantation is an interesting finding. Yet, the SCs in peripheral nerve fibers are capable of myelinating only one axon. We hypothesized that the SCs detected originate from oligodendrocyte precursors of the CNS. However, this requires further evidence.

Protein products of *mpz*, *pmp2*, and *pmp22* are myelin-associated proteins of the PNS. In particular, the protein PMP2 is a lipid transport protein of the SCs; it is also involved in myelination in the PNS. The protein PMP22 regulates the SC proliferation, the protein P0 (a *mpz* product) performs a stabilizing function for myelin layers relative to one another [38]. Although the protein products of these genes are highly specific for peripheral myelin, we detected low levels of the *mpz*, *pmp2*, and *pmp22* mRNA expression in the CNS also. The expression data obtained are consistent with the previous results. For example, it is shown that *pmp22* is expressed in motor neurons of the spinal cord and the brain, and *pmp2* and *mpz* are detected in oligodendrocytes [17, 39, 40]. The increased *pmp22* mRNA expression observed in the groups with of hUCB-MC transplantation might indicate an enhanced myelination of axons by the SCs, while its decrease in the SCI group indicating axonal degeneration [41, 42]. The data we obtained on the *pmp2* mRNA expression are consistent with the results of proteomic analysis of the spinal cord after SCI [43]. Positive shifts in the *mpz* mRNA expression indirectly indicate an increased SC number in the area of injury when hUCB-MCs are implanted.

Thus, the transplantation of hUCB-MCs, transduced with adenoviruses Ad5-VEGF/Ad5-GDNF or Ad5-EGFP into an area of the rat SCI, increases the number of SCs in the site of damage. The results obtained might indicate either increased migration of these cells and their proliferation, or decreased apoptosis in a focus of posttraumatic degeneration, which is to be clarified in subsequent analysis.

## Figures and Tables

**Table 1:** Primary and secondary antibodies used in immunofluorescent staining and Western blotting.

Antibody	Host	Dilution	Source
Krox 20	Goat	1 : 100 (IHC) 1 : 200 (WB)	Santa Cruz
Peripherin	Mouse	1 : 100 (IHC) 1 : 300 (WB)	Santa Cruz
P0	Mouse	1 : 50 (IHC) 1 : 50 (WB)	Santa Cruz
p75	Mouse	1 : 75 (IHC) 1 : 50 (WB)	Santa Cruz
Anti-goat IgG conjugated with Alexa 555	Donkey	1 : 200	Invitrogen
Anti-mouse IgG conjugated with Alexa 555	Donkey	1 : 200	Invitrogen
HRP-conjugated anti-goat IgG	Rabbit	1 : 200	Sigma
HRP-conjugated anti-mouse IgG	Rabbit	1 : 200	Sigma

**Table 2:** Primers and probes for RT-PCR.

Primer	Nucleotide sequence
Pmp22-TM-Forward	gTgCTAgTgTTgCTCTTC
Pmp22-TM-Reverse	GgATgTggTACAgTTCTg
Pmp22-TM-Probe	[FAM] CTCCACCATCgTCAgCCAAT [BH1]
Pmp2-TM-Forward	ggAgACTATATCACCATTAgA
Pmp2-TM-Reverse	TCCAgCAACTTTCTCTTTA
Pmp2-TM-Probe	[FAM]CCACTTCTgCACTTgCTTCA[BH1]
Mpz-TM-Forward	TCgCAAAgATgAgCAgAg
Mpz-TM-Reverse	ggCCCATCATgTTCTTgA
Mpz-TM-Probe	[FAM]CCAgTAGAACCAgCCTCAAgAAC[BH1]
Prx-TM-Forward	gAAgCCAAAgTAgTCAAgg
Prx -TM-Reverse	gggTTCCAggAgAgAAAg
Prx -TM-Probe	[FAM]CAgACTTCgAATgCCCACCTT[BH1]
18S–TM-Forward	gCCgCTAgAggTgAAATTCTTg
18S–TM-Reverse	CATTCTTggCAAATgCTTTCg
18S–TM-Probe	[HEX]ACCgCgCAAgACggACCAg[BH2]
